# *trans*-2-Enoyl-CoA Reductase Tecr-Driven Lipid Metabolism in Endothelial Cells Protects against Transcytosis to Maintain Blood-Brain Barrier Homeostasis

**DOI:** 10.34133/2022/9839368

**Published:** 2022-04-04

**Authors:** Jinxuan Wang, Jianxiong Xu, Guangchao Zang, Tao Zhang, Qi Wu, Hongping Zhang, Yidan Chen, Yi Wang, Weixi Qin, Shuang Zhao, Erdai Qin, Juhui Qiu, Xiaojuan Zhang, Lin Wen, Yeqi Wang, Guixue Wang

**Affiliations:** ^1^Key Laboratory for Biorheological Science and Technology of Ministry of Education, State and Local Joint Engineering Laboratory for Vascular Implants, Bioengineering College of Chongqing University, Chongqing 400030, China; ^2^Institute of Life Science, Laboratory of Tissue and Cell Biology, Lab Teaching & Management Center, Chongqing Medical University, Chongqing 400016, China; ^3^Chongqing Key Laboratory of Nano/Micro Composite Material and Device, School of Metallurgy and Materials Engineering, Chongqing University of Science and Technology, Chongqing 401331, China; ^4^College of Basic Medical Sciences, Chongqing Medical University, Chongqing 400016, China

## Abstract

The transport and metabolism of lipids in cerebrovascular endothelial cells (ECs) have been hypothesized to regulate blood-brain barrier (BBB) maturation and homeostasis. Long-chain polyunsaturated fatty acids (LCPUFAs) as the important lipids components of cell membranes are essential for the development and function of BBB, but the direct links of lipid metabolism and ECs barrier function remain to be established. Here, we comprehensively characterize the transcriptomic phenotype of developmental cerebrovascular ECs in single-cell resolution and firstly find that *trans*-2-enoyl-CoA reductase (Tecr), a very-long-chain fatty acid synthesis, is highly expressed during barriergenesis and decreased after BBB maturation. EC-specific knockout of Tecr compromises angiogenesis due to delayed vascular sprouting. Importantly, EC-specific deletion of Tecr loss restrictive quality of vascular permeability from neonatal stages to adulthood, with high levels of transcytosis, but maintains the vascular tight junctions. Moreover, lipidomic analysis shows that the expression of Tecr in ECs is associated with the containing of omega-3 fatty acids, which directly suppresses caveolae vesicles formation. These results reveal a protective role for Tecr in BBB integrity and suggest that Tecr as a novel therapeutic target in the central nervous system (CNS) diseases associated with BBB dysfunction.

## 1. Introduction

A healthy central nervous system (CNS) requires a homeostatic microenvironment and an integrated barrier to efficiently transport oxygen and nutrient, with free of bloodborne toxins [1, 2]. The blood-brain barrier (BBB) and blood-retina barrier (BRB) comprise a single layer of mature endothelial cells (ECs) that provide a continuous, nonfenestrated vascular system to separate the blood circulation and CNS. The development of the BBB is a gradual process, which begins with vascularization at embryo day 12. Then, the perineural vascular plexus will migrate to the brain in a conserved manner through sprouting angiogenesis [3, 4]. This early angiogenesis displays the acquisition of endothelial barrier properties, such as tight junction, influx, and efflux transporters. Although the brain has a more complex vascular structure than retina, retina also has a similarly gradual process in the formation of angiogenesis and barriergenesis [5]. This phenomenon signifies a major method to discover the development and related diseases of CNS.

ECs of the CNS are dramatically different compared with ECs in other tissues, which have three unique features to construct barrier for maintaining the CNS homeostasis [6]. First, endothelial tight junctions (TJs) prevent water-soluble molecules from circulating into the brain parenchyma though the paracellular passages. Second, ECs of the CNS exhibit a low rate of receptor-mediated transcellular trafficking, a phenomenon that assumed to limit EC transcytosis. Third, ECs as a first barrier limit immune cells entering into CNS, preventing neuroinflammation and pathology. While TJs are immediately acquired after ECs firstly enter the CNS, recent advances have highlighted that transcellular transport in ECs is assumed to regulate BBB permeability. Meanwhile, transcellular transport is activated in earlier developmental stages and gradually suppressed in mature stages [7]. Recent research also has revealed that the unique membrane lipid composition of CNS ECs regulates BBB permeability by suppressing caveolae-mediated transcytosis [8]. Moreover, emerging evidence reveals that ECs fatty acids (FAs) synthase influences ECs behavior and thus regulates angiogenesis [9, 10]. However, the complex function and morphology of lipid species in BBB are largely unknown. An understanding of how lipid metabolism induces BBB development and maintenance is not only to study BBB functions but also take advantages of diseases therapy and CNS drug delivery.

The development and homeostasis of brain depend on transfer and metabolism of FAs. Very long-chain fatty acids (VLCFAs) as chain-length of ≥22 carbons FAs are important for cell metabolism, membrane, and signaling transduction [11–13], which have a wide range of physiological functions particularly related to vascular permeability, inflammation, and neurocognitive function [14–16]. Tecr as the part of sphingosine 1-phosphate (S1P) metabolic pathway catalyzes the fourth step of the FAs elongation cycle and involves in both saturated and unsaturated VLCFAs [17–19]. In clinical trial, the mutation of *TECR* substituting Pro182 to Leu (P182L) causes autosomal recessive nonsyndromic mental retardation [18]. These findings suggest that Tecr drives saturation, and elongation process of VLCFAs may play an important role in regulating the function and structure of ECs. Yet, the mechanism of how VLCFA metabolism regulates endothelial function is still unclear.

Here, we firstly found that Tecr as a FAs synthetase was highly expressed in cerebrovascular ECs in early development stages. To determine whether and how Tecr affects vascular function and homeostasis in BRB and BBB, we have generated *Tecr*^iECKO^ mice and examined the formation and integrity of BRB/BBB during development. *Tecr*^iECKO^ mice showed BRB/BBB permeability as evidenced by high levels of endothelial transcytosis. Moreover, lipidomic analysis revealed that omega-3 FAs, which were direct suppressors of caveolae vesicles, were dramatically decreased after Tecr knockdown. Thus, this study demonstrated a new metabolic gene Tecr served as a key controller in omega-3 FAs metabolism and was essential for the maturation and maintenance of BRB and BBB.

## 2. Results

### 2.1. Single-Cell RNA-Sequencing (scRNA-Seq) Defines the Heterogeneity of Cerebrovascular ECs and Reveals that Tecr Expression Is Changed during BBB Development

**Figure 1:**
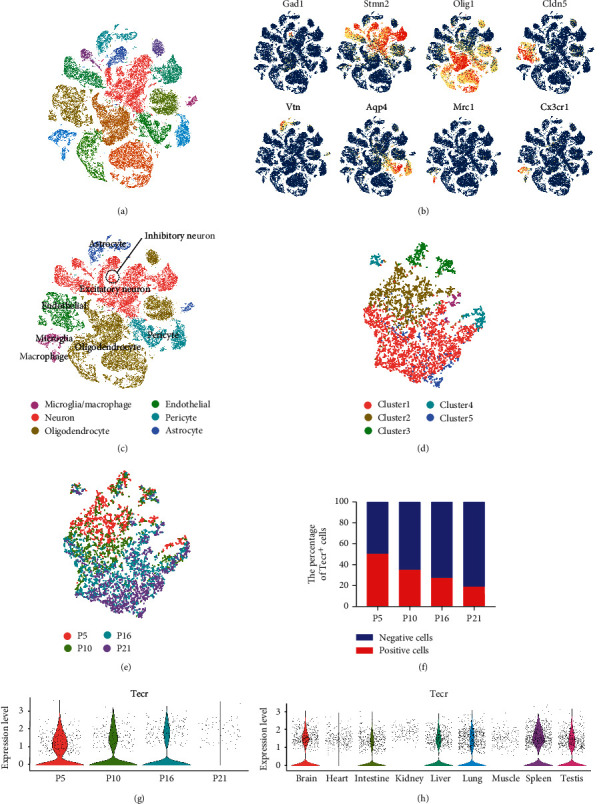
Transcriptomic characterization of P5-P21 LGN and Tecr expression. (a) t-SNE plot of cells extracted from P5, P10, P16, and P21 LGN. (b) Expression pattern of cluster markers defined different cell types. (c) t-SNE plot of cell types (inhibitory neuron, excitatory neuron, oligodendrocyte, endothelial, pericyte, astrocyte, macrophage, and microglia). (d) t-SNE plot of ECs subclusters. (e) t-SNE plot of ECs extracted from P5, P10, P16, and P21 LGN. (f) Proportions of Tecr^−^ (blue) and Tecr^+^ (red) cells for P5, P10, P16, and P21 LGN. (g) Violin plots of the expression of Tecr at P5, P10, P16, and P21 stages' ECs. (h) Violin plots of the expression of Tecr in the indicated tissues' ECs.

Brain vascular barriergenesis is a multistep process, which occurs during both embryonic and postnatal development. Mouse retina as an elegant model is widely used to study the development of BBB. During the postnatal life, the superficial capillaries of mice will sprout in early P6, form the intermediate vascular plexus during P10-P15, and fully form in P21 [20]. To lineage trace brain vascular EC development, we analyzed brain dorsal lateral geniculate nucleus (LGN) scRNA-Seq datasets GSE108761 provided by Kalish et al. [21] and scRNA-Seq datasets E-MTAB-8077 of ECs from multiple mouse tissues provided by Kalucka et al. [22]. The LGN scRNA-Seq datasets contained 57226 cells that passed quality filtering (P5: 19270 cells, P10: 11873 cells, P16: 16540 cells, P21: 9543 cells). Subsequently, Seurat was utilized to build graph-based clusters and group cells based on the Euclidean distance in PCA space [23]. Then, data was displayed through t-distributed stochastic neighbor embedding (t-SNE) plots with 17 different clusters (Figure 1(a)). Next, we annotated cell populations on the basis of published markers, including inhibitory neuron (Gad1^+^), excitatory neuron (Stmn2^+^), oligodendrocyte (Olig1^+^), endothelial (Cldn5^+^), pericyte (Vtn^+^), astrocyte (Aqp4^+^), macrophage (Mrc1^+^), and microglia (Cx3cr1^+^) (Figures 1(b) and 1(c)).

To further investigate the heterogeneity of ECs during BBB development, we selected Cdh5^+^ Cldn5^+^ EC cluster at P5, P10, P16, and P21. ECs from different development stages exhibited prominent transcriptomic heterogeneity. P5 and P10 stages ECs were distinct from P21 ECs, which are differently located on cluster1 and cluster2 (Figures 1(d) and 1(e)). Thus, we compared the diversity of gene expression and found that the reduction of Tecr^+^ cells was observed at development stages with 51% at P5 versus 19% at P21 (Figure 1(f)). To characterize the expression of Tecr in the ECs from different stages, we measured the relative expression of cells from each group and found Tecr was dramatically decreased at P21 (Figure 1(g)). Furthermore, in order to discovery the expression level of Tecr in ECs of different tissues, the scRNA-Seq datasets from Kalucka et al. [22], which included 39,182 ECs from 9 main tissues of adult mouse, were selected to analyze. We found that Tecr was abundantly expressed in spleen, lung, and brain (Figure 1(h), Figure S1a). Meanwhile, we also analyze the scRNA-Seq datasets from the Tabula Muris Consortium [24], containing 14483 cells from brain, heart, kidney, liver, lung, and muscle with 24 clusters and 16 cell types (Figure S2a). Then, CD31^+^ ECs were selected from different organs and exhibited prominent transcriptomic heterogeneity (Figure S2b). We also found that Tecr was abundantly expressed in brain (Figure S2c-d). Further immunohistology analysis also revealed that Tecr was obvious colocalization with blood vessels marker zonula occludens-1 (Zo-1) in mouse brain (Figure S1b). Thus, we supposed that the expression of Tecr was critical for ECs to form functional barrier during BBB development. Meanwhile, the decreasing expression of Tecr at P21 might be due to the BBB maturation.

### 2.2. Endothelial Tecr Plays a Promoting Role in Sprouting Angiogenesis

Forming a functional BRB/BBB firstly requires coordination of tissue-specific angiogenesis and EC differentiation [25]. To examine the effect of Tecr on EC phenotypic modulation during retinal and cerebrovascular vessels developing, we generated an EC-specific conditional *Tecr* knockout mice (*Tecr*^iECKO^ or KO) by crossing *Tecr*^fl/fl^ mice with *VE-cadherin-CreER^T2^* mice (Figure S3a). These mice were identical at the EC^lin^ mice, except at *Tecr* locus where both *Tecr* alleles were flanked with LoxP sites (*Tecr^ΔEC/ΔEC^*). When tamoxifen was administered, the *Tecr* gene was permanently deleted in all ECs. Real-time quantitative PCR (RT-qPCR) analysis of primary ECs isolated from *Tecr*^iECKO^ mice showed 70% knockout efficiency of Tecr (Figure S4a). Flox/flox-positive but *VE-cadherin–CreER^T2^*-negative mice among the littermates for each experiment were defined as wild-type (WT) mice. Three baseline doses of tamoxifen were administered to mice from postnatal day 1 (P1) to P3, and retinas were analyzed at P5 and P7 (Figure S3a). The *Tecr*^iECKO^ mice at P7 showed impaired retinal angiogenesis (Figure S3b and Figure S5a–c) and exhibited reduced outgrowth, vascular density, and branching in the retina, with 17%, 33%, and 29% reduction, respectively, compared to WT mice (Figure S3c–e). Moreover, tip ECs at the vascular front of *Tecr*^iECKO^ mice exhibited reduced sprouting numbers, but no difference in the numbers of filopodia at the vascular front (Figure S3f–h). Furthermore, in order to study whether defect angiogenesis is due to the decrease of proliferative ECs, we administered phospho-histone H3 (PHH3^+^) labeling (red) to assess the number of proliferative ECs in retinas. *Tecr*^iECKO^ mice showed no difference in the number of IB4^+^PHH3^+^ ECs compared to WT (Figure S3i–j). These findings indicated that loss of endothelial Tecr caused vascular defects by impairing EC migration but not proliferation.

### 2.3. Blood-Retinal Barrier is Broken Down in *Tecr*^iECKO^ Mice

**Figure 2:**
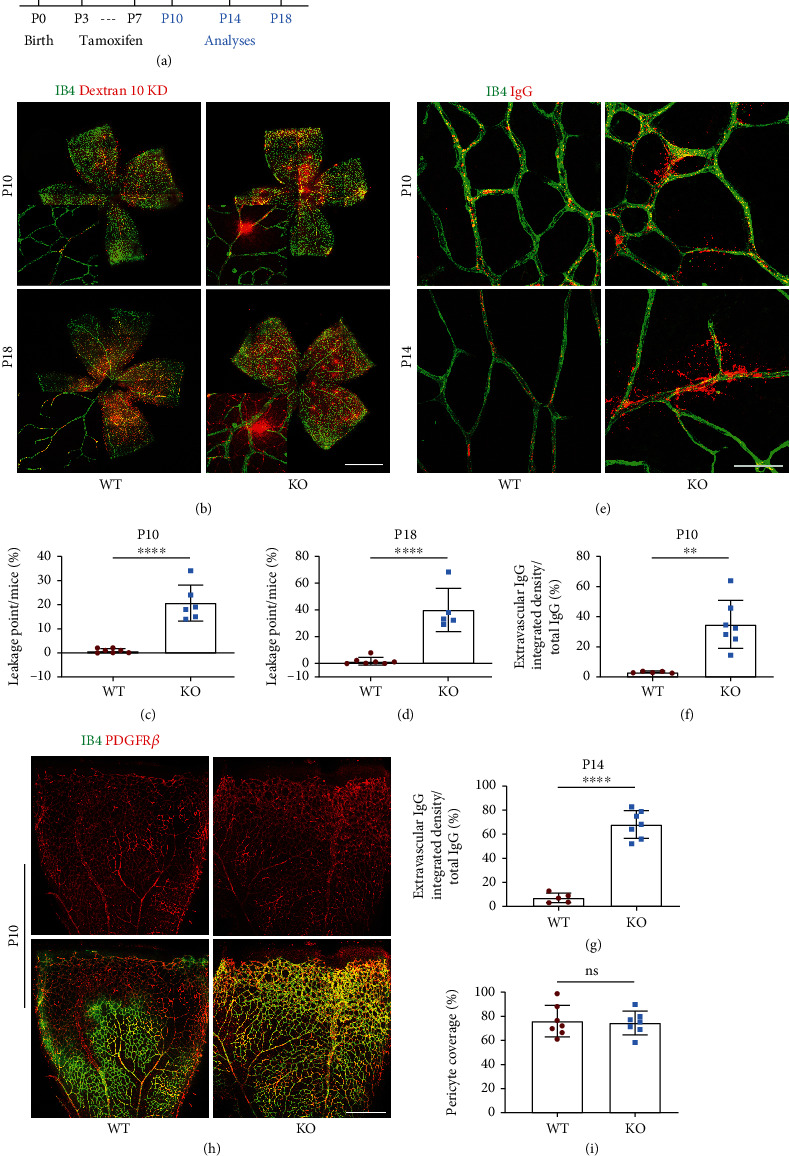
Endothelial Tecr was essential for BRB maturation. (a) Diagram depicting the experimental schedule for EC-specific deletion of *Tecr* from p3 and analysis at P10, P14, or P18. (b) Confocal images of whole retinal vasculature of the WT and *Tecr*^iECKO^ mice after injected 10 kDa dextran at P10 and P18. ECs, IB4 (green); dextran (red). Scale bars, 2 mm. (c, d) Leakage points of WT and *Tecr*^iECKO^ retinas at P10 (c) and p18 (d). The leakage points were measured by the number of dextran^+^ IB4^−^ area. *n* ≥ 5 mice per group. *p* < 0.0001, Unpaired *t*-test. (e) Confocal images of retinal vasculature of the WT and *Tecr*^iECKO^ mice after injected IgG at P10 and P14. Scale bars, 40 *μ*m. (f, g) Permeability index WT and *Tecr*^iECKO^ retinas at P10 (f) and p14 (g). The permeability index is measured by the ratio of IgG^+^ IB4^−^ area over IgG^+^ area. *n* ≥ 5 mice per group. (f) *p* = 0.0013; (g) *p* < 0.0001; unpaired *t*-test. (h) Representative images of Pdgfr*β*^+^ (red, pericyte) coverage onto IB4^+^ (green) vessels in WT and *Tecr*^iECKO^ retinas at P10. Scale bars, 400 *μ*m. (i) Quantitative analysis of pericyte coverage onto vessels in WT and *Tecr*^iECKO^ retinas. *n* = 7 per group. ns: not significant. Data are expressed as mean ± SEM.

The maturation of vascular barrier and angiogenesis is coordinated and simultaneous during cerebrovascular system formation [26]. During barriergenesis, functional BRB is fully acquired by P10, which has specialized TJs and low-rated transcytosis [7]. For further determining the role of Tecr in BRB formation, five baseline doses of tamoxifen were administered to *Tecr^ΔEC/ΔEC^* mice from P3 to P7 (Figure 2(a)), and the retinas were analyzed at P10. *Tecr*^iECKO^ mice exhibited no significant difference on retinal density compared to WT mice (Figure S6a–b). Furthermore, we examined whether loss of endothelial Tecr affects vascular barrier maturation in retina. To measure BRB permeability, we injected the fluorescein conjugated-dextran (10-KD) through left ventricle at P10 and P18 and measured its distribution and levels within the retina after 10-minute (mins) circulation. Excitingly, the dextran tracer permeated in the parenchyma, indicating the leakage of the BRB in *Tecr*^iECKO^ mice (Figures 2(b)–2(d)). To further confirm the maturation of BRB, we studied whether plasma-derived proteins accumulate in the tissue parenchyma of BRB in the *Tecr*^iECKO^ mice. Plasma-derived immunoglobulin G (IgG) was injected to P10 and P14 mice, revealing significant perivascular IgG deposits in *Tecr*^iECKO^ mice compared with WT (Figure 2(e)). Quantitative analysis showed 20-fold and 30-fold increase in IgG accumulation in the tissue parenchyma of P10 and P14 *Tecr*^iECKO^ mice compared to WT, respectively (Figures 2(f)–2(g)). These leaks were randomly distributed in the retinas, regardless of distal or proximal in *Tecr*^iECKO^ mice. Recent studies have reported that the support of pericytes to ECs plays a vital role in maintaining BBB integrity [3, 27, 28]. Therefore, we also examined the possibilities that Tecr regulates EC barrier function was related to pericytes coverage. Nevertheless, we found that there were no differences in coverage of Pdgfr*β*^+^ pericytes onto ECs between *Tecr*^iECKO^ and WT mice at P10 retinal vessels (Figures 2(h) and 2(i)). Thus, endothelial Tecr was critically involved in the maturation of BRB in an EC-autonomous manner.

### 2.4. Endothelial Tecr Is Required for Maturation and Maintenance of the BBB

**Figure 3:**
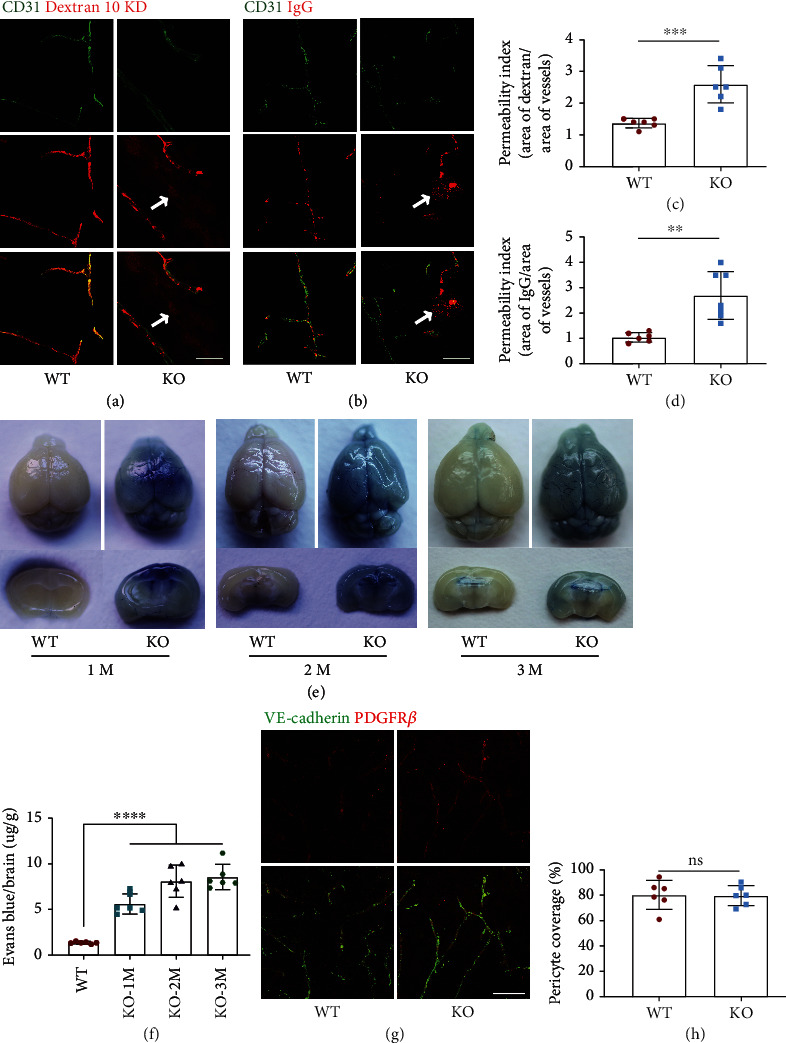
Loss of Tecr functions in ECs caused BBB breakdown. (a, b) *Tecr*^iECKO^ mice displayed BBB leakage. 10 kD dextran (red) and IgG tracer (red) is completely confined within vessels (green, CD31) in WT mice. Tracer-filled parenchyma cells (arrows) surround vessels in the *Tecr*^iECKO^ mice brain. Scale bar, 50 *μ*m. (c, d) Permeability index of tracer leakage in brain, as quantified by area of tracer divided by area of vessels per image (value = 1 signifying no leakage). *n* ≥ 6 mice per group. (c) *p* = 0.0006; (d) *p* = 0.0015; unpaired *t*-test. (e) Evans blue examination of BBB at 1 month, 2 months and 3 months. Evans blue examination showed that the BBB integrity was disrupted in *Tecr*^iECKO^ mice. *n* = 6 mice per group. (f) Quantification of Evans blue staining showed leakage in the brain parenchyma at 1 month, 2 months, and 3 months in *Tecr*^iECKO^ mice. *n* = 6 mice per group. *p* < 0.0001; unpaired *t*-test. (g) Representative images of Pdgfr*β*^+^ (red, pericyte) coverage onto IB4^+^ cerebrovascular vessels in 2 months WT and *Tecr*^iECKO^ mice. The brain was coronal sectioned. Every section is 15 *μ*m. Scale bars, 105 *μ*m. (h) Quantitative analysis of pericyte coverage onto cerebrovascular vessels in WT and *Tecr*^iECKO^ mice brains. *n* = 6 mice per group. ns not significant. Data are expressed as mean ± SEM.

Since loss Tecr induced vascular abnormalities in the retina and a breakage in BRB, we further evaluated whether Tecr is also target for BBB maturation at P10. To measure BBB permeability, we administered tamoxifen as previous scheme (Figure 2(a)). Fluorescein conjugated-dextran (10-KD) and IgG were injected in left ventricle at P10. The distribution and levels of both fluorescent dyes were measured after 15 min circulation in brain. We found that *Tecr*^iECKO^ mice have significant difference of tracer leakage compared to WT. High-resolution confocal microscopy confirmed that the dextran and IgG were accumulated around capillary ECs in *Tecr*^iECKO^ mice brain (Figures 3(a)–3(d)). These phenomena suggested that endothelial Tecr was required for maturation of BBB.

The development of mice brain is fully completed at postnatal 1 month [16]. Tecr was expressed in adult mice brain (Figure S1a). Therefore, we assumed whether Tecr also plays a role in maintaining vascular integrity during the whole lifetime of mice. To prove this conjecture, five baseline doses of tamoxifen were administered to *Tecr^ΔEC/ΔEC^* mice. Two weeks after tamoxifen injection, Evans blue was injected into 1-, 2-, and 3-month old mice heart. As expected, we found that the permeability of cerebrovascular vessels in *Tecr*^iECKO^ mice to Evans blue staining was significantly increased, while almost no leakage in WT mice (Figures 3(e) and 3(f)). Consistent with previous results, there was no significant difference in the coverage of Pdgfr*β*^+^ pericytes onto ECs in the cerebral vessels (Figures 3(g) and 3(h)), indicating that the function of Tecr was EC-autonomous during lifetime. Thus, these results revealed that Tecr not only regulated vascular barrier maturation but also maintained BBB stability.

### 2.5. The Transcellular but Not Paracellular BRB/BBB Permeability Occurs in *Tecr*^iECKO^ Mice

**Figure 4:**
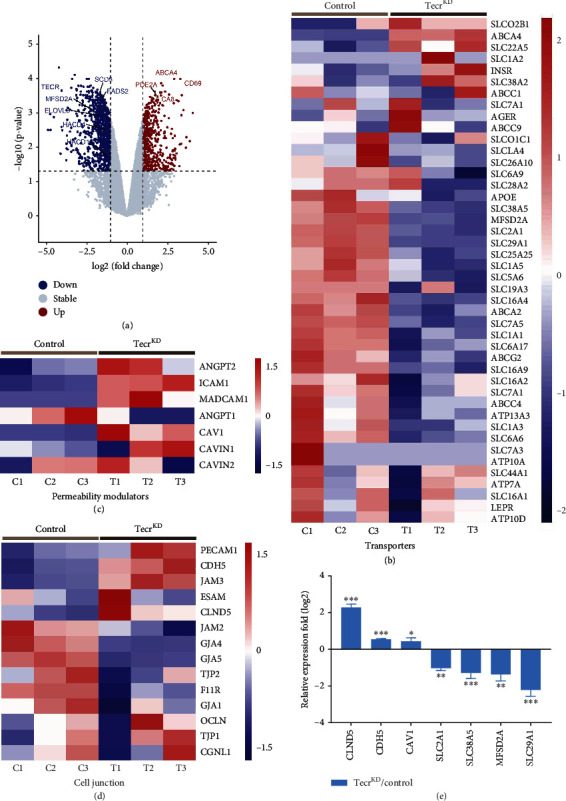
Key signaling pathways governed the barrier function. (a) Volcano plots were used to display the magnitude of the differential expression between control and Tecr^KD^ ECs. *n* = 3 per group. (b)–(d) Heatmap showed the expression level of EC transporters-related genes (b), permeability-related genes (c), and cell-to-cell junction-related genes (d), compared with control and Tecr^KD^ ECs. (e) Representative of BBB-related genes from transcriptome analysis was reconfirmed by RT-qPCR. ^∗∗∗^*p* < 0.001, ^∗∗^*p* < 0.01, ^∗^*p* < 0.05. Data are expressed as mean ± SEM. Unpaired Student's *t*-test.

To define the contribution of endothelial Tecr on vascular leakage of the barrier, we further carried out gene expression profiles analysis of primary human umbilical vein endothelial cells (HUVECs) from Tecr siRNA knockdown (Tecr^KD^) and control. Transcriptome analysis identified that Tecr^KD^ ECs caused a total of 508 upregulation genes and 1035 downregulation genes (*p* < 0.05) (Figure 4(a), Figure S4b–d). Among the RNA-Seq analysis, we found that a broad range of BBB-related transporter genes were significantly downregulated in Tecr^KD^ ECs, including *Mfsd2a*, *Slc2a1*, *Slc7a1*, *Slc7a5*, *Slc1a1*, *Slc38a5*, and *Abcg2* (Figure 4(b)). Importantly, Tecr knockdown also resulted an increasing expression of vascular permeability genes, including *Angpt2*, *Icam1*, and *Madcam1*, indicating that vascular permeability is increased. Meanwhile, there was no difference on Angpt1, a Tie2 receptor ligand that is known to decrease permeability (Figure 4(c)). We then further discovered the expression and activation of two unique pathways, including transcytosis and paracellular flux, which are important in regulating barrier permeability. Tecr^KD^ ECs exhibited dramatical changes in the expression of transcytosis-related genes (*Cav-1*) (Figure 4(c)). Whereas there were upregulation on TJs (*Tjp1*, *Ocln*, and *Clnd5*) and adheren junction (*Cdh5*) genes in Tecr^KD^ ECs (Figure 4(d)). These findings were reconfirmed by RT-qPCR (Figure 4(e)). Overall, these results indicated a significant increase in transcytosis pathway in Tecr^KD^ ECs.

Because EC barrier preventing paracellular flux relies on the presence of EC junctions, we reconfirmed the expression of Zo-1, Claudin5, and VE-cadherin by western blotting, showing upregulation in Tecr^KD^ ECs (Figure S7). To verify these insights in vivo, we confirmed the BRB-forming junction proteins, Claudin-5, and VE-cadherin showed similar localization and expression at cell junctions in the superficial and deep plexi of p10 and p18 *Tecr*^iECKO^ and WT mice retinas (Figure S8a–f). Furthermore, we also examined the expression and subcellular localization of VE-cadherin and Zo-1 at adult brains, showing no difference in both of *Tecr*^iECKO^ and WT mice (Figure S9a–c). These in vitro and in vivo results indicated that EC barrier leakage was independent of paracellular flux.

**Figure 5:**
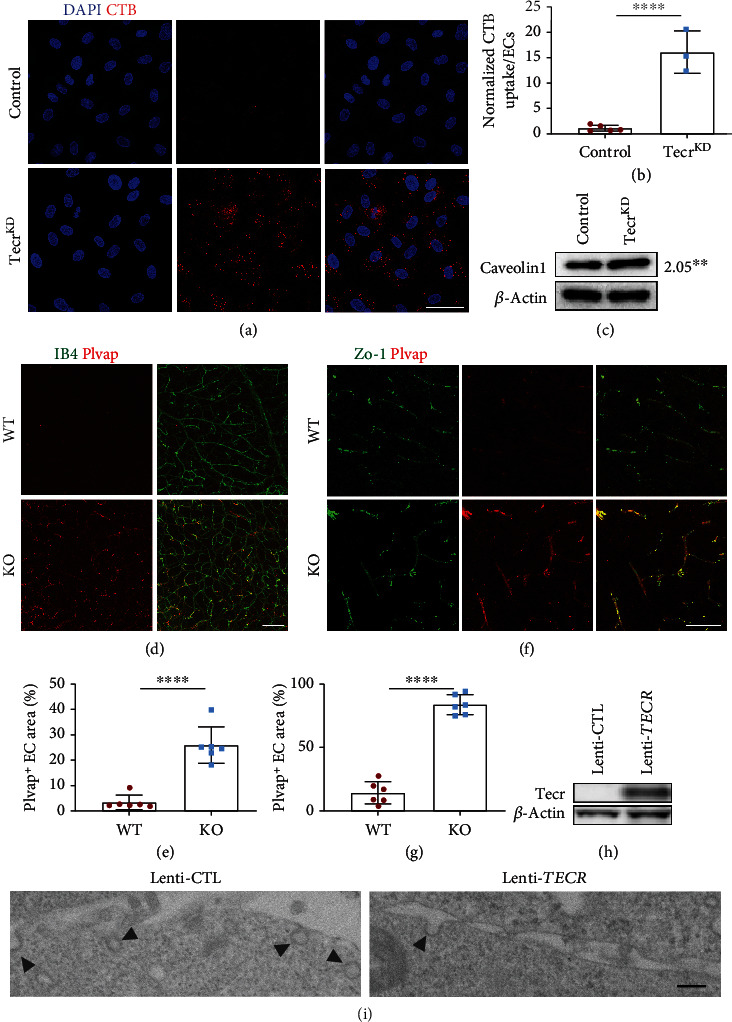
Loss of Tecr increased transcellular permeability. (a) Tecr^KD^ ECs showed significantly enhanced uptake activity of CTB compared with control. CTB, red; DAPI, blue. Scale bars, 50 *μ*m. (b) Quantitation of CTB uptake per cell. *n* = 5 control and *n* = 3 Tecr^KD^. *p* = 0.0002, unpaired *t*-test. (c) Western blot analysis and quantification of Cav-1 in control and Tecr^KD^ ECs. Tecr^KD^ ECs showed a dramatic increase in Cav-1. *p* = 0.0059, unpaired *t*-test. (d) Immunofluorescence images for Plvap of the WT and *Tecr*^iECKO^ retinas at P18. Plvap was highly expressed in the *Tecr*^iECKO^ retinas. Scale bars, 80 *μ*m. (e) The expression of Plvap is significantly increased in *Tecr*^iECKO^ retinas at P18. *n* = 6 per group. *p* < 0.0001, unpaired *t*-test. (f) Immunofluorescence images for Plvap of the WT and *Tecr*^iECKO^ brains at 2 months. Scale bars, 70 *μ*m. (g) The expression of Plvap is significantly increased in *Tecr*^iECKO^ brains at 2 months. *n* = 6 per group. *p* < 0.0001, unpaired *t*-test. (h) Lenti-*TECR* was successfully expressed in Hela cells. (i) Electron microscope images showed the apical plasma membrane of Hela cells after overexpressing human *TECR*. Overexpressing *TECR* showed reducing vesicular pit density (arrows) in Hela cells, compared to mock-infected control. Scale bars, 4 *μ*m. Data are expressed as mean ± SEM.

Since the junctions of ECs had no defect after Tecr knockdown, transcytosis as another defining feature of EC barrier was considered to be regulated by Tecr. To further confirm that Tecr regulated EC transcytosis, we developed a fluorescent cholera toxin subunit B- (CTB-) based transcytosis assay in vitro. We found that Tecr^KD^ ECs exhibited a significant increase of CTB uptake compared to control in HUVECs and human cerebral microvascular endothelial cells (hCMECs) (Figures 5(a) and 5(b), Figure S10a–c). During vesicle trafficking of EC, there are two major transcytosis pathways, including clathrin-mediated transcytosis and caveolae-mediated transcytosis. Interestingly, Caveolin1 (Cav-1) as an important member of caveolae has been reported to be involved in endothelial vesicles' trafficking and formation [29, 30]. RNA-seq analysis showed an increased expression of *Cav-1*, but no difference on *clathrin*, indicating that Tecr played an important role in modulating caveolae-mediated transcytosis (Figure 4(c)). Then, we verified the expression of Cav-1 by western blot (Figure 5(c)).

Furthermore, recent reports have demonstrated that Plvap, which is located on fenestrae and caveolar stomatal diaphragms, is essential for vesicle formation, transportation, and its decrease is important for BBB maturation with low-rate transcytosis [31]. Meanwhile, the interactions between pericytes and ECs are essential for the expression of Plvap in brain [3, 32–37]. Thus, we stained WT and *Tecr*^iECKO^ littermates and found that Plvap was continuously and highly expressed in *Tecr*^iECKO^ P18 retina (Figures 5(d) and 5(e)) and adult mice brain (Figures 5(f) and 5(g)). To further assess whether Tecr is sufficient to suppress endocytic vesicle formation, Hela cell lines which have no endogenous Tecr expression were utilized to establish a lentiviral overexpression cell line (Figure 5(h)). Electron microscope images revealed a reduction in the number of vesicles after overexpressing Tecr (Figure 5(i)). These data revealed that Tecr was important for regulating transcellular trafficking, but not paracellular, in regulating barrier property of brain and retina.

### 2.6. Tecr Knockdown Results in Aberrant Lipid Homeostasis

**Figure 6:**
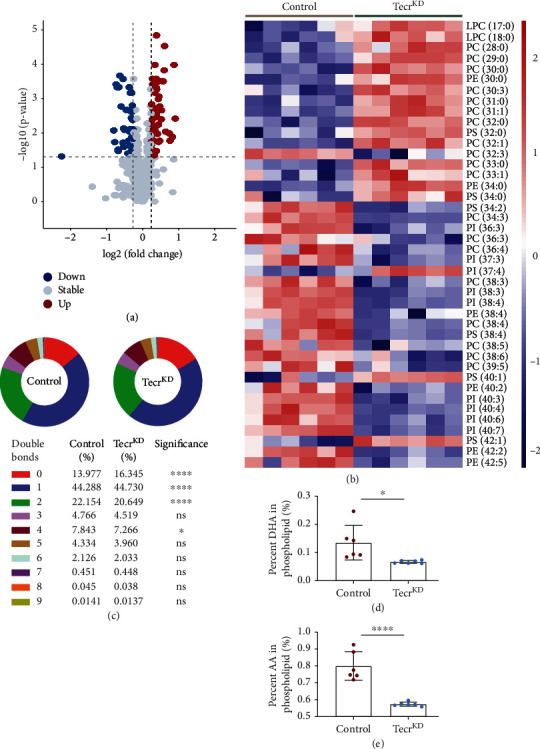
Effect of Tecr knockdown on synthesis and transport of phospholipids in primary HUVECs. (a) The volcano plots visualized the different lipid molecules between control and Tecr^KD^ ECs. *n* = 6 per group. (b) The effect of Tecr on elongating FAs was analyzed by targeted lipidomic between control and Tecr^KD^ ECs. (c) The effect of Tecr on desaturating FAs was analyzed by targeted lipidomic between control and Tecr^KD^ ECs. ^∗∗∗∗^*p* < 0.0001, ^∗^*p* = 0.0264, unpaired *t*-test. (d, e) Total DHA levels (d) and total AA (e) levels in control and Tecr^KD^ ECs. DHA and AA levels were expressed as mean ± SEM of the percentage of total phospholipids. (d) *p* = 0.022; (e) *p* < 0.0001; unpaired *t*-test. Data are expressed as mean ± SEM.

In our previous findings, we have demonstrated that *Tecr*^iECKO^ mice exhibited BBB permeability, suggesting an increasing transcytosis and accelerating caveolae-based endocytic vesicle formation. Tecr as a reductase involved in VLCFAs synthesis and VLCFAs' saturation step, which drives the reprogramming of lipid metabolism [19]. To determine how Tecr affects BBB functions, we further performed lipidomic profiling in Tecr^KD^ ECs and determined 381 unique lipid molecules of 18 analyzed lipid classes. The volcano plots demonstrated that 90 lipids were significantly changed with 54 upregulation and 36 downregulation (Figure 6(a)). Remarkably, heatmap also demonstrated a profound decreasing in VLCFAs during Tecr knockdown (Figure 6(b)). These results were consistent with the characteristic that Tecr was involved in the production of VLCFAs on ECs. Moreover, another biologic function of Tecr was to partially mediate the catalysis of FAs saturation. Therefore, data were transformed to pie chart containing the percentages of the bonds per sample for each lipid, showing a significant decrease of 2 or more double bonds in the combined phosphatidylcholine, phosphatidylethanolamine, phosphatidylinositol, and phosphatidylserine pools in Tecr^KD^ ECs (Figure 6(c)). On the other hand, RNA-seq was utilized to analyze the expression of metabolism-related genes regulating fatty acid desaturases *Δ*9, *Δ*6, and *Δ*5 during de novo synthesis [38, 39]. Supporting our previous results, knockdown Tecr significantly downregulated desaturation step-related genes, such as SCD, SCD5, FADS1, and FADS2 (Figure S11). These results indicated that Tecr could regulate lipid metabolism by inducing changes in desaturation-related genes. These results showed that Tecr not only plays an important role in the synthesis of VLCFAs but also in the transformation of VLCFAs and long-chain polyunsaturated fatty acids (LCPUFAs). Thus, we further focused on the brain most abundant LCPUFAs: docosahexaenoic acid (DHA) and arachidonic acid (AA), which were important for endothelial transcytosis in BBB [8, 40]. Furthermore, DHA enrichment in endothelial membrane induces a high level of membrane fluidity and causes a replacement of cholesterol and Cav-1, resulting in caveolae formation inhibition [7, 8, 41, 42]. Lipidomic analysis revealed a significant 45% reduction in DHA-containing phospholipids and 28% reduction in AA-containing phospholipids in Tecr^KD^ ECs (Figures 6(d) and 6(e)). Therefore, these results suggested that Tecr affected the formation of caveolae through regulating the DHA metabolism in ECs.

## 3. Discussion

More and more studies have shown that endothelial lipid metabolism is necessary for brain and visual system, especially in the development and function of blood vessels. Many researchers have discovered that LCPUFAs especially DHA metabolism is associated with the maintenance and formation of BBB, whereas only a few numbers of reports have characterized the metabolic pathways in ECs, especially in how EC lipid metabolism regulates vascular remodeling and barrier integrity [16, 43, 44]. The current evidence from the recent outbreak supports that the synthesis and reaction of FAs, as a prime regulator in metabolite signaling, contribute to explaining the process of angiogenesis [45, 46]. However, the role of lipid metabolism in ECs remains incompletely characterized. Here, we showed that loss of the endothelial Tecr impaired lipid metabolism resulting transcytosis-mediated BRB/BBB breakdown. Our findings support a model in which Tecr-mediated formation of caveolae vesicles regulates the maturation and maintenance of BRB/BBB (Figure S12). These findings provide direct evidence that endothelial lipid metabolism plays an important role in BRB/BBB functions.

### 3.1. Loss of Endothelial Tecr Impairs BBB Integrity Due to Disordered Transcytosis

EC dysfunction due to disordered transcytosis in the brain results in extensive CNS vascular barrier defects and aberrant BBB functions [3, 27, 47]. LCPUFA known as the important components of cell membrane is a type of transcytosis controller predominantly regulating the integrity of BBB [8, 48, 49]. Meanwhile, FAs especially LCPUFAs metabolism leads to interrupted migration, proliferation, and sprouting in ECs [50–52]. The question of how and whether the metabolism of lipid in ECs can regulate membrane LCPUFA components to mediated BBB formation has yet to be examined. Our results demonstrated that loss of Tecr in ECs could result an abnormal leakage on brains and retinas from child to adult. RNA-seq results and consistent of in vivo and vitro experiments pointed to the ability of Tecr to induce a dramatically change in transcytosis, with increase of vascular permeability, but no defect on TJs. In general, Tecr is sufficient to suppress endocytic vesicle formation, which maintains a low transcytosis rate of ECs. Recently, Yang et al. report that an age-related shift BBB dysfunction which transforms ligand-specific receptor transcytosis to nonspecific transcytosis and shows a dramatical difference in lipid composition [53]. However, the relationship between lipid metabolism and BBB dysfunction is still unclear.

### 3.2. Knocking Down Tecr Results in Reduced DHA Accumulation

Although loss of Tecr induced increased transcytosis and resulted BRB/BBB leakage, it is still unknown how Tecr regulates composition of membrane to facilitate the caveolae-based vesicles across into ECs membrane. Previous studies have reported that Tecr as a lipid metabolism gene is involved in VLCFAs synthesis and catalyzes the step of saturation [17], resulting abnormal LCPUFA content in brain [8, 54]. Whereas it is still unclear what caused the changes of lipid metabolism and resulting BBB leakage. In our study, we firstly found a profound decrease in the total amount of VLCFAs and composition of 2 or more double bonds. FAs in Tecr^KD^ ECs, consistent with the dramatical changes in fatty acid elongation and desaturation, catalyze step-related gene (Figure S11). These data suggested that Tecr potentially regulates LCPUFA metabolism. Recently, Clark et al. show that omega-3 phospholipid species especially DHA in brain ECs are important for maintaining the low rate of transcytosis [55]. Jabs et al. report that the abnormal lipid metabolism could influence the trans-endothelial transport of FAs [52]. Meanwhile, DHA also could alter caveolae microenvironment not only by modifying membrane lipid composition but also by changing the distribution of major structural proteins [42]. Gu et al. also point to a direct role of DHA in the suppression of caveolae vesicles [8]. In agreement, the levels of DHA and AA were reduced in our results. These findings indicate that Tecr may regulate the lipids composition of ECs to command transcytosis. Future investigations will enable us to detect the molecular mechanisms by which Tecr regulates DHA metabolism.

Together, we report an unknown metabolism pathway whereby Tecr as a FAs synthetase affects the BBB homeostasis through regulating endothelial transcytosis. Meanwhile, we discover the importance of the Tecr as a “commander” of the endothelial LCPUFA metabolism. Given the special role of Tecr-driven lipid metabolism induces BBB homeostasis, our data may serve as a key therapeutic clue of CNS drug delivery and desire sites for repairing BBB dysfunction.

## 4. Materials and Methods

### 4.1. Analysis of scRNA-Seq Data

ScRNA-seq data were screened using the GEO (http://www.ncbi.nlm.nih.gov/geo) datasets GSE108761 [21] and ArrayExpress (https://www.ebi.ac.uk/arrayexpress) datasets E-MTAB-8077 [22]. Raw data were processed using R (version 3.8.3). The following quality process and analysis were utilized the R package Seurat [23, 56]. Genes expressed by less than 3 cells were filtered and discarded. Then, the cells were selected by 500 < expressing genes < 5000 genes and containing <7.5% mitochondrial genes. Data were normalized by using the NormalizeData function, which raw genes counts from each cell were normalized to the whole counts. The resulting expression data was then scaled with log-transformed and summarized by principal component analysis (PCA) with graph-based clustering method. t-SNE plot was used for victualing the resulting clusters in two-dimension. Cell clusters were classified based on the expression of known marker genes, including inhibitory neuron (Gad1^+^), excitatory neuron (Stmn2^+^), oligodendrocyte (Olig1^+^), endothelial (Cldn5^+^), pericyte (Vtn^+^), astrocyte (Aqp4^+^), macropahge (Mrc^+^), and microglia (Cx3cr1^+^).

### 4.2. Mouse Models and Inducible Genetic Experiments

Specific pathogen-free (SPF) *Tecr* floxed mice (*Tecr^fl/fl^*) were generated by BRL^+^ medicine (Shanghai, China). Tamoxifen-inducible EC-specific *Tecr* mice (*Tecr^ΔEC/ΔEC^*) were generated by crossing *Tecr*^fl/fl^ with *VE-cadherin–CreER^T2^* driver. Mice were established, fed in standard SPF animal environment with free growth and reproduction feed and water. PCR primers for the *Tecr^fl/fl^* allele are forward GTGCCACTCGGGTTACATC and reverse CGAATCTCCACCTCAAAGAA. PCR primers for the *VE-cadherin–CreER^T2^* are forward TTCCCGCAGAACCTGAAGATG and reverse CTACACCAGAGACGGAAATCCATC. To induce Cre activity in the *Tecr^ΔEC/ΔEC^* mice, 50 *μ*l (1 mg/ml in ethanol/corn oil) tamoxifen was injected intraperitoneally to P1-3 or p3-7 mice. The phenotypes of *Tecr*^iECKO^ mice and their littermates were analyzed at P5-7 or P10-18. For Evans blue experiment, tamoxifen was injected intraperitoneally for 5 consecutive days from the indicated time point [57]. Both male and female mice were used in all experiments, and no gender differences were observed. Animals were carried out with review and approval from the Laboratory Animal Welfare and Ethics Committee of Chongqing University.

### 4.3. Tissue Isolation and Immunofluorescent

Retinas were harvested from WT and *Tecr*^iECKO^ mice as described previously [57]. In brief, mice were perfused with phosphate-buffered saline (PBS) and 4% paraformaldehyde (PFA). Tissue was fixed for 15 min at room temperature and washed with PBS. Whole-mount retinas were dissected to isolate the retinal cup, and relieving cuts were made prior to staining. Brains were fixed by immersion in 4% PFA/PBS overnight at 4°C, dehydrated in 30% sucrose, and then frozen in TissueTek OCT (Sakura). After fixation, retinas or brain sections were blocked for 2 h in 10% goat serum/5% BSA with 0.5% Triton X-100 and incubated overnight with the following antibodies: isolectin B4, Alexa Fluor 647-conjugate (Thermo Fisher Scientific, 1 : 100), rat anti-VE-cadherin (BD Biosciences, 1 : 250), rat anti-CD31 (Abcam, 1 : 200), rat anti-Tecr (Thermo Fisher Scientific, 1 : 100), rat anti-Plvap (BD Biosciences, 1 : 200), mouse anti-Claudin-5, and Alexa Fluor 488-conjugated (Thermo Fisher Scientific, 1 : 200). For detection, suitable species-specific Alexa Fluor-coupled secondary antibodies (1 : 500) were used. Tissues were imaged using confocal microscopy (Leica).

### 4.4. BBB Permeability Assay

#### 4.4.1. Postnatal

P10-P18 pups were anaesthetized with isoflurane and fluorescent tracer 10 kDa dextran tetramethylrhodamine (Thermo Fisher Scientific, Cat #D1817, 10 mg/ml, 10 *μ*l per g body weight) or IgG (Jackson ImmunoResearch, Cat #715-165-150, 1.25 mg/ml, 5 *μ*l per g body weight) was injected into the left-ventricle with 31 gauge, 0.3 cc insulin syringe. The heartbeat was monitored for a steady heartbeat that was continuous for appropriate time. After circulation, 10 min of dextran and 20 min of IgG, retinas were harvested and fixed using methods as previously described, costained with isolectin B4 or Claudin-5 to visualize blood vessels.

#### 4.4.2. Adult

200 *μ*l of Evans blue (2%; Aicon Biotech) was injected into adult mice by cardiac perfusion. The mice were transcardially perfused after 10 min circulation, and then the brains were dissected. The tissues stained with Evans blue dye were homogenized in 1 ml of PBS, sonicated, and centrifuged for 30 min (15,000 rpm). The supernatant was collected, adding 500 *μ*l of 50% trichloroacetic acid for 12 hours at 4°C, and then centrifuged (30 min, 15,000 rpm, 4°C). Evans blue staining was measured by Colibri (Titertek-Berthold) at 610 nm.

#### 4.4.3. Quantitative Analysis of the Retinal Vasculature

All quantifications were done on high-resolution confocal images representing a thin z section of the sample. The number of branchpoints and the area covered by ECs were calculated with the ImageJ software. The number of endothelial sprouts and filopodial extensions was quantified at the angiogenic front of control (WT) and *Tecr*^iECKO^ retinas. PHH3-labelled isolectin B4-positive ECs were counted. Data are based on a minimum of three independent experiments or three mutant and control animals for each stage and result shown.

#### 4.4.4. Endothelial Cell Culture and RNA Interference

Primary HUVECs purchased from ScienCell were maintained in endothelial cell medium supplemented with low serum growth supplement (ScienCell). Primary HUVECs at passage 5-6 was used for experiments. Primary HUVECs seeded on 1-5 *μ*g/cm^2^ bovine plasma fibronectin (ScienCell) coated flasks were transfected with siRNAs by using Lipofectamine™ RNAiMAX (Thermor fisher scientific) according to the manufacturer's instructions. The following target sequences were used: human Tecr #1 (5′-GGAUCGGUUUCGCCAUCAUTT-3′) and #2 (5′-AUGAUGGCGAAACCGAUCCTT-3′) (GenePha Kalucka rma). Efficiency of knockdown was evaluated by RT-qPCR and western blotting. Experiments were started at 48 h after transfection.

#### 4.4.5. RNA-Seq

The RNA-Seq was performed on three independent biological replicates. According to manual instruction, total RNA was extracted from the HUVECs using Trizol (Takara). About 1∗10^6^ cells were ground into powder by liquid nitrogen in a 1.5 mL tube, followed by being homogenized for 2 min and rested horizontally for 5 min. The mix was centrifuged and then the supernatant was transferred into a new EP tube with 0.3 mL chloroform/isoamyl alcohol (24 : 1). The mix was centrifuged again after mixed well. The upper aqueous phase where RNA remained was transferred into a new tube with equal volume of supernatant of isopropyl alcohol and then centrifuged. After deserting the supernatant, the RNA pellet was fully washed with 75% ethanol. Finally, RNA was dissolved in 50 *μ*L DEPC water. Subsequently, total RNA was qualified by Nano Drop (Thermo Fisher Scientific). Purified mRNA was fragmented into small pieces. Then, first-strand cDNA was generated using random hexamer-primed reverse transcription, followed by a second-strand cDNA synthesis. Then, A-Tailing Mix and RNA Index Adapters were added. The cDNA fragments which were dissolved in EB solution were amplified and purified. The quality of product was validated by the Agilent Technologies 2100 bioanalyzer. The double-stranded PCR products were processed to get the final library. The single-strand circle DNA (ssCir DNA), as the final library, was amplified with phi29. DNA nanoballs (DNBs) were loaded into the patterned nanoarray. Finally, single end 50 bases reads were generated on BGIseq500 platform (BGI-Shenzhen, China).

#### 4.4.6. Western Blotting

Protein lysate samples were boiled at 95°C, run on 4-15% polyacrylamide gels, and electrophoretically transferred onto PDVF membranes. Membranes were blocked with 5% nonfat milk/TBST for 1 h and incubated overnight at 4°C with the following primary antibodies: VE-cadherin (Santa Cruz, 1 : 500), Zo-1 (Invitrogen, 1 : 500), beta-actin (Cell Signaling Technology, 1 : 1000), Tecr (Bethyl laboratories, 1 : 1000), Cav-1 (NOVUS, 1 : 1000), and Claudin-5 (Thermo Fisher Scientific, 1 : 1000). Membranes were then incubated with the appropriate HRP-conjugated secondary antibody (Proteintech, 1 : 5000) and developed with regular ECL (Thermo Fisher Scientific). The intensity of individual bands was quantified using ImageJ.

#### 4.4.7. Transmission Electron Microscopy

Hela were transfected with PLVX-IRES-Puro lentiviral plasmid containing full-length human *TECR* CDS. Cells experiments on Hela cells were performed 7 days postinfection with lentivirus. The Hela cells in the culture flask were scraped off by cell scraper. The cell suspension was collected into the tube and centrifuged at 1500 rpm for 15 min. Discarded the supernatant and slowly added 0.5% glutaraldehyde. Left at 4°C for 10 min and centrifuged at 12000 rpm for 15 min. Discarded the supernatant and added 3% glutaraldehyde. Cultures were processed for TEM.

#### 4.4.8. In Vitro Cholera Toxin Subunit-B Uptake Assays

Uptake procedure was adapted from a combination of previously described protocols [8]. Confluent primary HUVECs were transfected with Tecr siRNA by Lipofectamine™ RNAiMAX. Tecr knockdown was performed with lentivirus (Shanghai Genechem Co., Ltd.) on hCMECs according to the manufacturer's instructions. Cultures were then incubated with cholera toxin subunit-B (CTB) Alexa-555 conjugate (Thermo Fisher Scientific, Cat #C34776, 150 mg/ml) for 10 min at 37°C, washed several times with cold PBS, fixed in 4% PFA/PBS for 15 min at room temperature, washed with PBS, and mounted with Antifade Mounting Medium for imaging.

#### 4.4.9. Lipidomic Analysis

After the culture of primary HUVECs, the culture medium was removed. Washed with PBS buffer for 3 times quickly, removed PBS buffer, and scraped off adherent cells with cell curets. The collected cells were transferred into 1.5 mL EP tubes, which were quick-frozen with liquid nitrogen and transported with dry ice. Lipid extraction and detection were performed by BGI-Shenzhen, China. Levels of individual phospholipid species were analyzed as described previously [58].

#### 4.4.10. Image Acquisition, Processing, and Analysis

Confocal microscopy was performed (Leica). For image analysis, ImageJ software was used. The Figs were processed and assembled using Adobe Photoshop and Adobe Illustrator. The only adjustments used in the preparation of the Figs were for brightness and contrast. For comparison purposes, different sample images of the same antigen were acquired under constant acquisition settings.

#### 4.4.11. Statistical Analysis

A standard software package (GraphPad Prism, version 6) was used to all statistical analyses. Data obtained for the two groups of mice or cells were expressed here as mean values ± standard error of the mean (±SEM). One-way ANOVA followed by two-tailed Student's test was used to reveal the differences between the groups. Significant differences between groups were set at ∗*p* < 0.05, ∗∗*p* < 0.01, ∗∗∗*p* < 0.001, and ∗∗∗∗*p* < 0.0001.

## Additional Points

*Significance Statement*. Using single-cell RNA sequencing, we firstly found that endothelial cells (ECs) in differently developmental stages exhibit a dramatical change of Tecr expression. Selective deletion of endothelial Tecr affected blood-brain barrier (BBB) maturation and homeostasis through disrupting endothelial transcytosis. Moreover, using lipidomic analysis, we further identified that the intrinsic changes in endothelial transcytosis are due to the reduction of docosahexaenoic acid and arachidonic acid. These data serve as a key protective role for Tecr-driven lipid metabolism in BBB homeostasis and provide a therapeutic clue for BBB disorder and central nervous system (CNS) drug delivery.

## Supplementary Materials

Supplementary Materials S1: Tecr was highly expressed in cerebrovascular vessels. Figure S2: the expression of Tecr in ECs of different tissues. Figure S3: loss of Tecr functions in ECs caused vascular defects at P7. Figure S4: Tecr was efficiently knocked down in vivo and in vitro. Figure S5: Tecr had no effect on retinal growth at P5. Figure S6: Tecr had no effect on retinal vasculature density at P10. Figure S7: loss of endothelial Tecr did not impair cell junctions. Figure S8: loss of endothelial Tecr did not impair cell junctions in retinas. Figure S9: loss of endothelial Tecr did not impair cell junctions in brains. Figure S10: knockdown of Tecr in hCMECs significantly enhanced uptake activity of CTB. Figure S11: knockdown of Tecr significantly altered the expression pattern of lipid metabolism pathway. Figure S12: model for the suppression of caveolae-mediated transcytosis via regulated DHA-containing in phospholipid at the ECs of BRB/BBB.
