# Supplementary material for: trans-2-Enoyl-CoA Reductase Tecr-Driven Lipid Metabolism in Endothelial Cells Protects against Transcytosis to Maintain Blood-Brain Barrier Homeostasis
Source: Research (Wash D C). 2022 Apr 4;2022:9839368. doi: 10.34133/2022/9839368 (PMC9006154; doi:10.34133/2022/9839368)
Supplement: Supplementary Materials — S1: Tecr was highly expressed in cerebrovascular vessels. Figure S2: the expression of Tecr in ECs of different tissues. Figure S3: loss of Tecr functions in ECs caused vascular defects at P7. Figure S4: Tecr was efficiently knocked down in vivo and in vitro. Figure S5: Tecr had no effect on retinal growth at P5. Figure S6: Tecr had no effect on retinal vasculature density at P10. Figure S7: loss of endothelial Tecr did not impair cell junctions. Figure S8: loss of endothelial Tecr did not impair cell junctions in retinas. Figure S9: loss of endothelial Tecr did not impair cell junctions in brains. Figure S10: knockdown of Tecr in hCMECs significantly enhanced uptake activity of CTB. Figure S11: knockdown of Tecr significantly altered the expression pattern of lipid metabolism pathway. Figure S12: model for the suppression of caveolae-mediated transcytosis via regulated DHA-containing in phospholipid at the ECs of BRB/BBB. [file 9839368.f1.docx]

**Supplementary Materials**


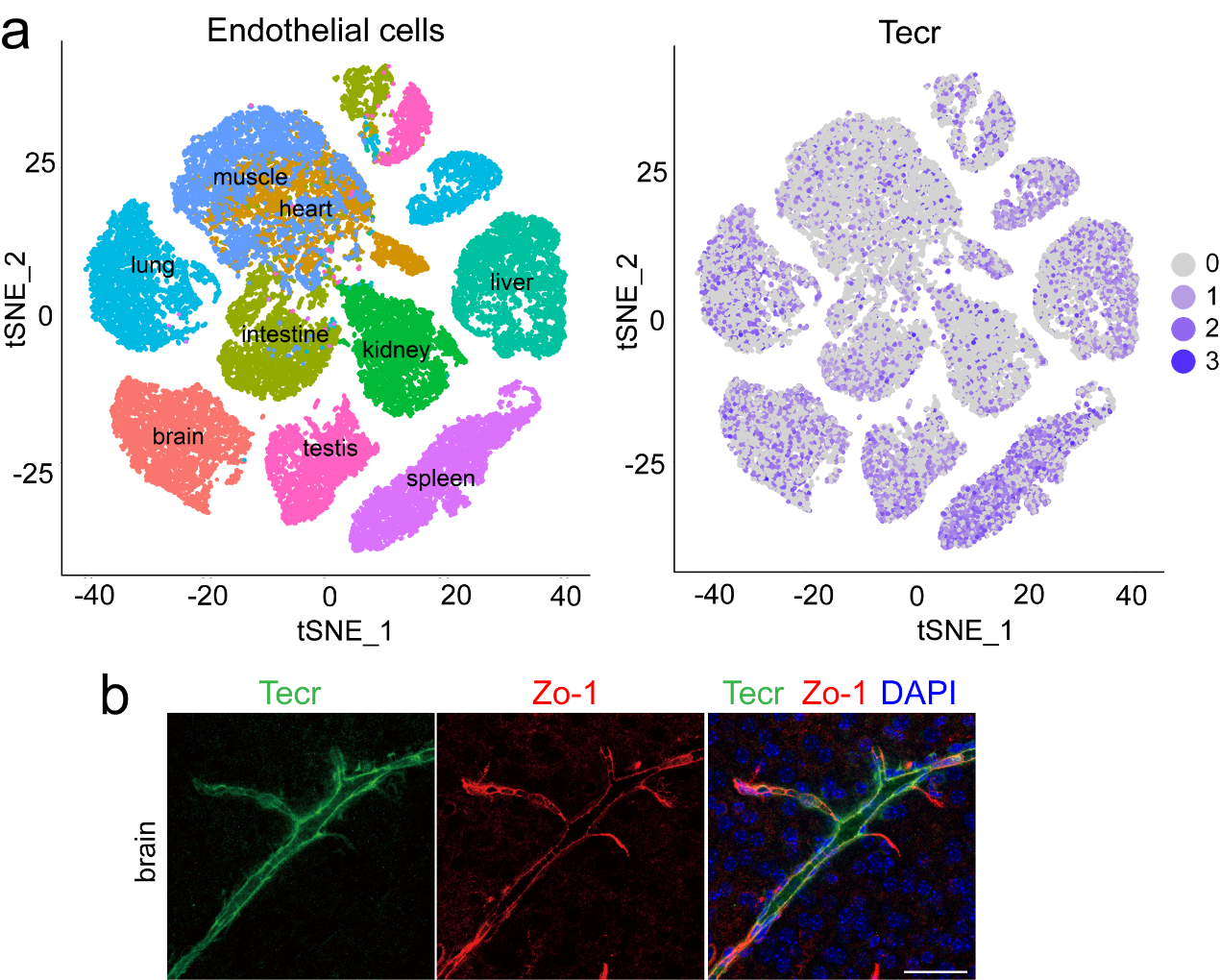


**Fig. S1. Tecr was highly expressed in cerebrovascular vessels.**

a. Tecr expression in ECs from multiple tissues was analyzed by scRNA-Seq. Tecr was highly expressed in lymphatic and cerebrovascular vessels.

b. The expression of Tecr was detected by immunofluorescence in cerebrovascular vessels of mice at P5. Tecr (green) has obvious co-localization onto Zo-1^+^ (red) vessels. Scale bars, 50 μm.


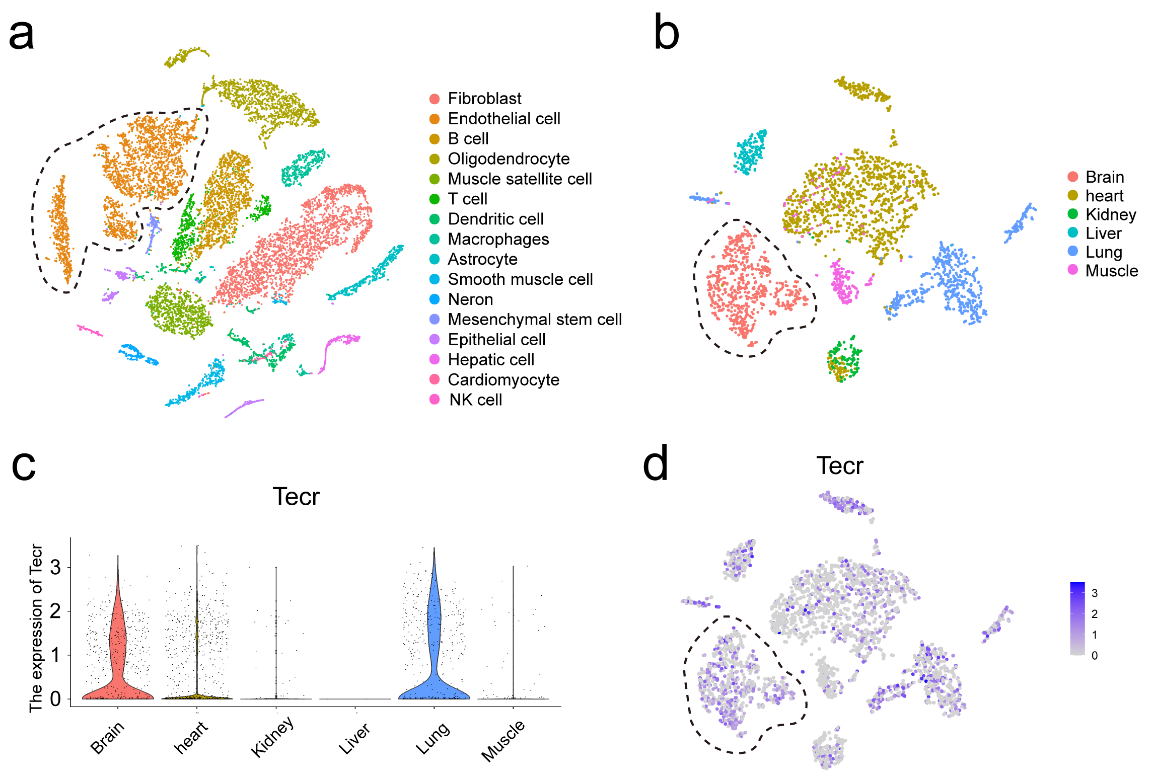


**Fig. S2. The expression of Tecr in ECs of different tissues.**

a. t-SNE plot of cells extracted from mice brain, heart, kidney, liver, lung, muscle.

b. t-SNE plot of ECs sub-clusters.

c. Violin plots of the expression of Tecr from mice brain, heart, kidney, liver, lung, muscle’s ECs.

d. Tecr expression in ECs from multiple organs was analyzed by scRNA-Seq.


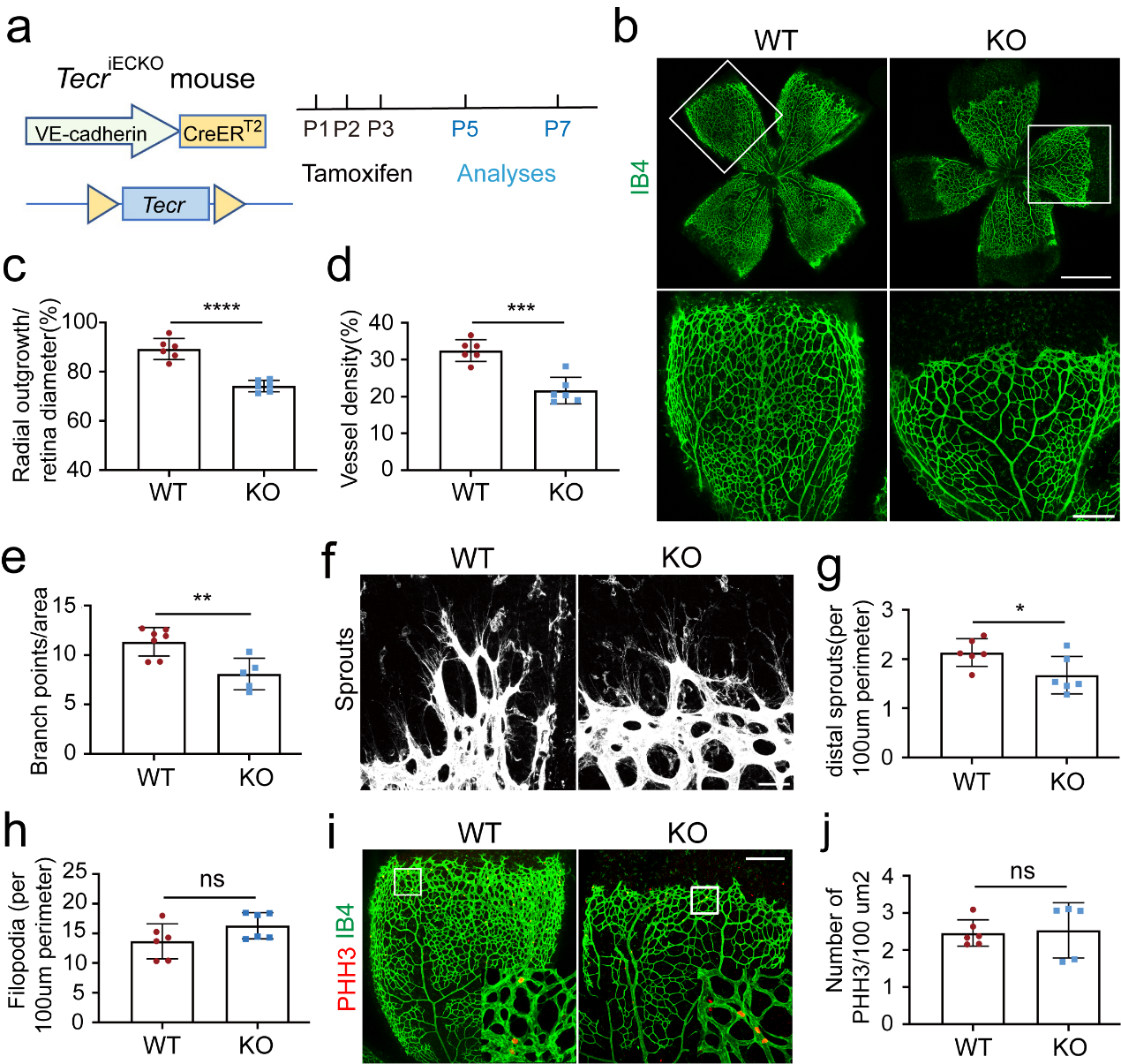


**Fig. S3. Loss of Tecr functions in ECs caused vascular defects at P7.**

a. Diagram depicting the schedule of *Tecr^ΔEC/ΔEC^* mice experiments for EC-specific deletion of *Tecr* from p1 and analysis at P5 or P7.

b. Representative images of P7 retinal vascular plexuses with IB4 staining from WT and *Tecr*^iECKO^ mice. The insets (white dashed-line boxes) of up panel are magnified in down panel. Scale bars, upper, 2 mm; bottom, 450 μm.

c-e. Quantitative analysis of radial outgrowth (c), vessel density (d) and branch points (e) in retinal vasculature of WT and *Tecr*^iECKO^ mice. n≥5 mice per group. c, *p* < 0.0001; d, *p* = 0.0002; e, *p* = 0.0042; Unpaired t test.

f. Appearance of the P7 vessel growth front in WT and *Tecr*^iECK^*^O^* mice. Scale bars, 60 μm.

g-h. Quantitative analysis of distal sprouts (g) and filopodia (h) at the retinal vessels front of WT and *Tecr*^iECKO^ mice. n=6 mice per group. *p* = 0.0378, Unpaired t test.

i. Representative images of IB4 (green)^+^ PHH3 (red)^+^ proliferative ECs in retinal vessels of P7 WT and *Tecr*^iECKO^ mice. Scale bars, 450 μm.

j. Quantitative analysis of PHH3^+^ IB4^+^ proliferative ECs at the front of the retinal vessels. n≥5 mice per group.

ns not significant. Data are expressed as mean ± SEM.


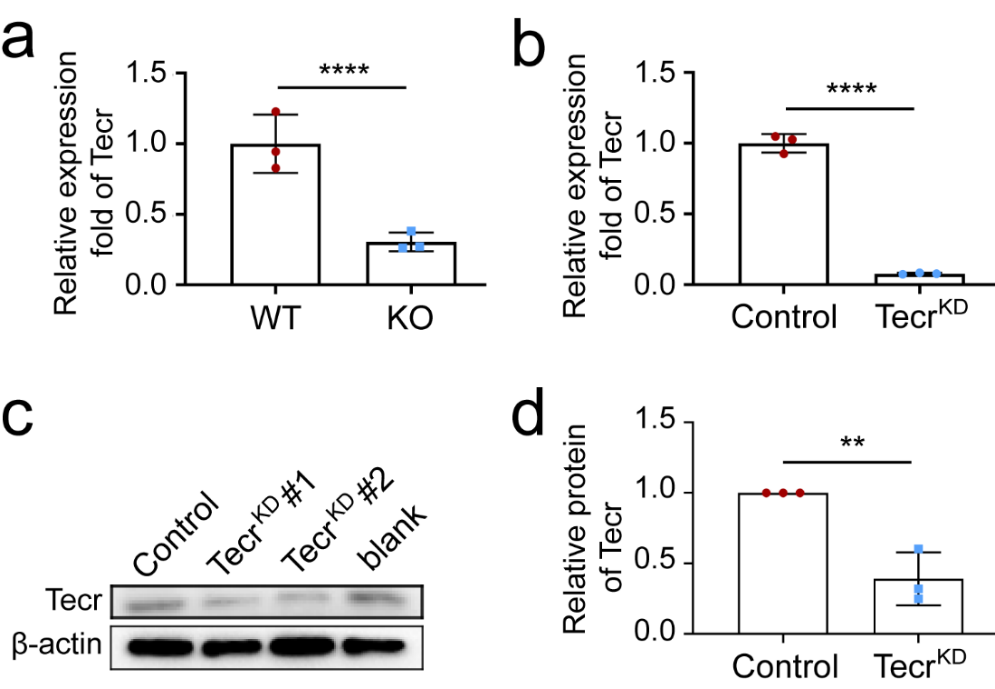


**Fig. S4. Tecr was efficiently knocked down in vivo and in vitro.**

a. RT-qPCR analyzed the expression of Tecr in *Tecr*^iECKO^ and WT mice. The *Tecr* knockout efficiency induced by tamoxifen was about 80%. *p* < 0.0001, Unpaired t test.

b. RT-qPCR analyzed the expression of Tecr in Tecr^KD^ ECs. The knockdown efficiency of Tecr in Tecr^KD^ ECs was 90%. *p* < 0.0001, Unpaired t test.

c. Western blot analyzed the expression of Tecr in control and Tecr^KD^ ECs. Control, treated with control RNA interference. Tecr^KD^, treated with Tecr RNA interference. Blank, treated with only Lipofectamine™ RNAiMAX.

d. Protein levels of Tecr in Tecr^KD^ ECs were quantified and normalized by β-actin levels. *p* = 0.0049, Unpaired t test.

Data are expressed as mean ± SEM.


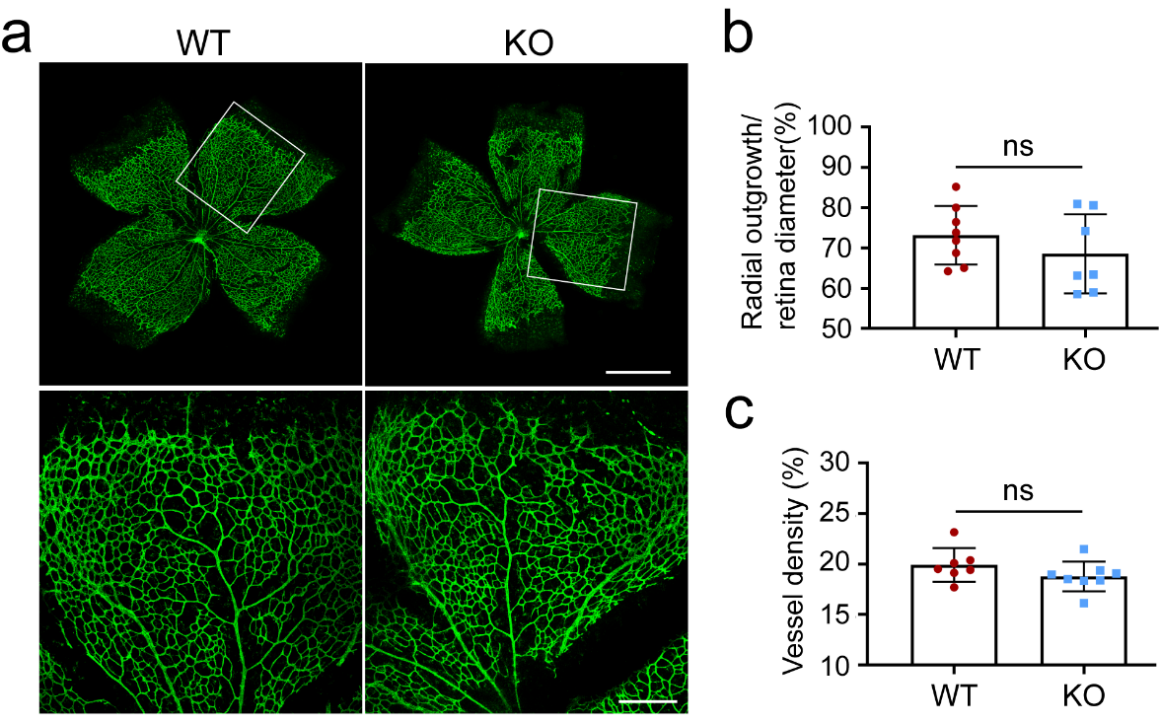


**Fig. S5. Tecr had no effect on retinal growth at P5.**

a. Representable images of IB4 staining retinal vasculature of WT and *Tecr*^iECKO^ mice at P5. Scale bars, above, 2 mm; bottom, 450 μm.

b. Quantification of radial outgrowth of the retinal vasculature. No significant differences were observed. n ≥7 mice per group.

c. Quantification of vessel density of the retinal vasculature. No significant differences were observed. n ≥7 mice per group.

ns not significant. Data are expressed as mean ± SEM. Unpaired t test.


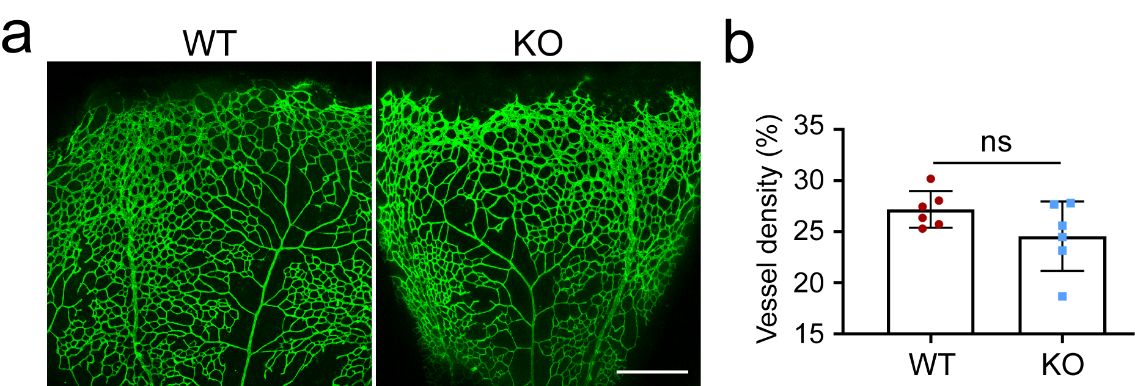


**Fig. S6. Tecr had no effect on retinal vasculature density at P10.**

a. IB4 staining of retinal vascular plexuses from WT and *Tecr*^iECKO^ mice at P10. Scale bars, 350 μm.

b. Quantification of vessel density of the retinal vasculature. No significant differences were observed. n = 6 per group.

ns not significant. Data are expressed as mean ± SEM. Unpaired t test.


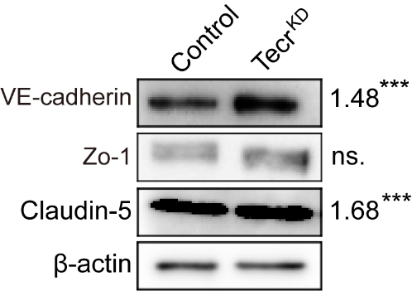


**Fig. S7. Loss of endothelial Tecr did not impair cell junctions.**

Western blot analysis and quantification of adheren junction protein VE-cadherin and TJs proteins Zo-1 and Claudin-5 in control and Tecr^KD^ ECs.

^***^*p* < 0.001, ns not significant. Data are expressed as mean ± SEM. Unpaired t test.


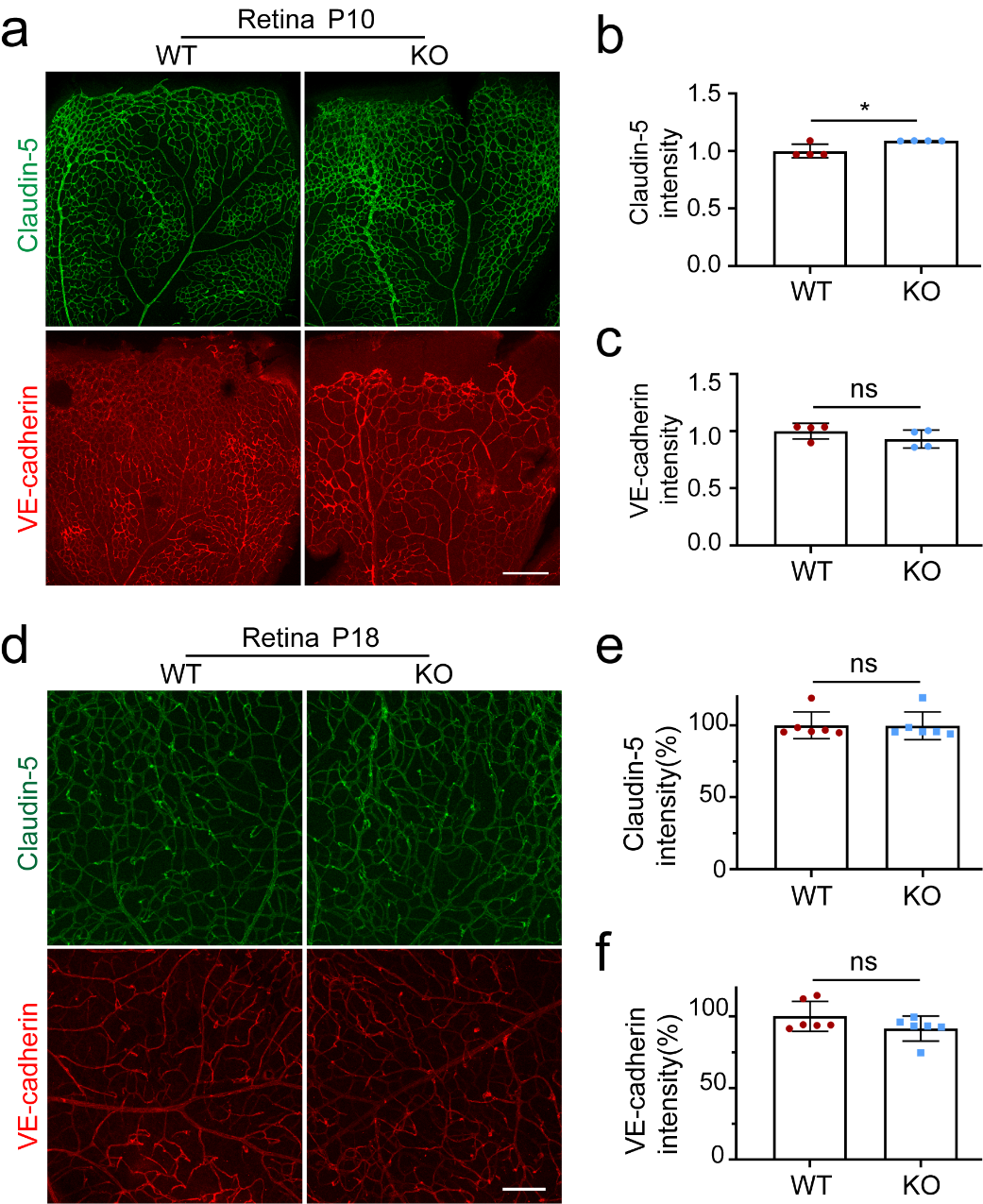


**Fig. S8. Loss of endothelial Tecr did not impair cell junctions in retinas.**

a. Immunofluorescence images for Claudin-5 (green) and VE-cadherin (red) in WT and *Tecr*^iECKO^ retinas at P10. Claudin-5 and VE-cadherin were localized to cell junctions in both genotypes. Scale bars, 450 μm.

b-c. Quantitative analysis of Claudin-5 (b) and VE-cadherin (c) expression showed no significant defect between WT and *Tecr*^iECKO^ retinas at P10. b, *p* = 0.0257, Unpaired t test.

d. Immunofluorescence images for Claudin-5 (green) and VE-cadherin (red) of the WT and *Tecr*^iECKO^ retinas at P18. Claudin-5 and VE-cadherin were localized on cell junctions in both genotypes. Scale bars, 80 μm.

e-f. Quantitative analysis showed no difference on Claudin-5 (e) and VE-cadherin (f) expression levels between WT and *Tecr*^iECKO^ retinas at P18. n = 6 per group.

ns not significant. Data are expressed as mean ± SEM. Unpaired t test.


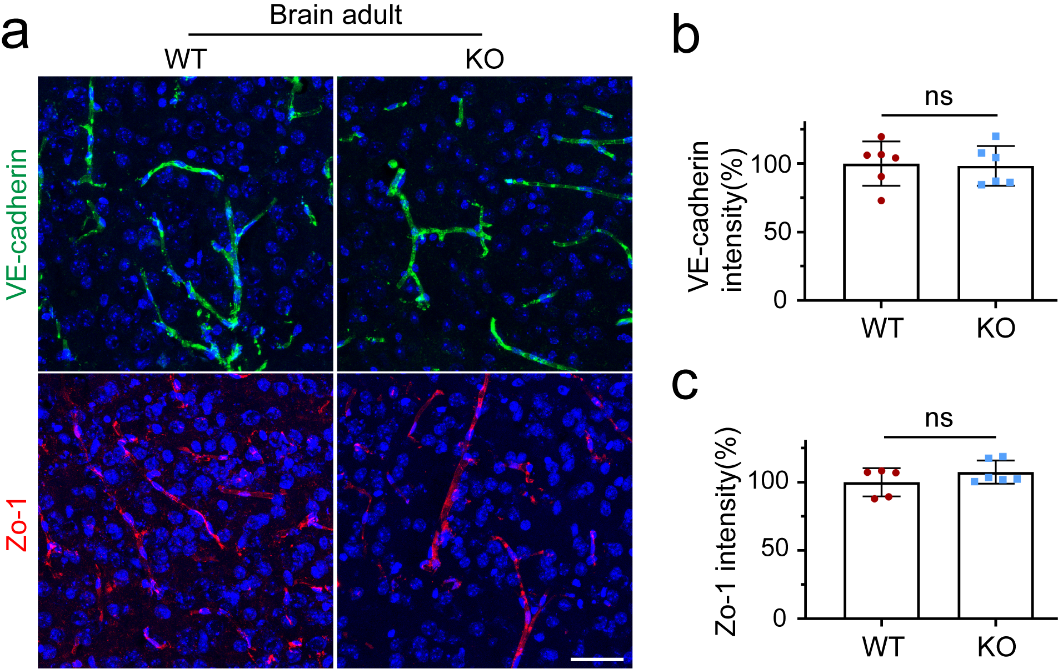


**Fig. S9. Loss of endothelial Tecr did not impair cell junctions in brains.**

a. Immunofluorescence images of VE-cadherin (green) and Zo-1 (red) in the WT and *Tecr*^iECKO^ brains at 2 months. VE-cadherin and Zo-1 were localized on cell junctions in both genotypes. Scale bars, 70 μm.

b-c. Quantitative analysis shows equal VE-cadherin (b) and Zo-1 (c) expression levels between WT and *Tecr*^iECKO^ brains at 2 months. n ≥ 5 per group.

ns not significant. Data are expressed as mean ± SEM. Unpaired t test.


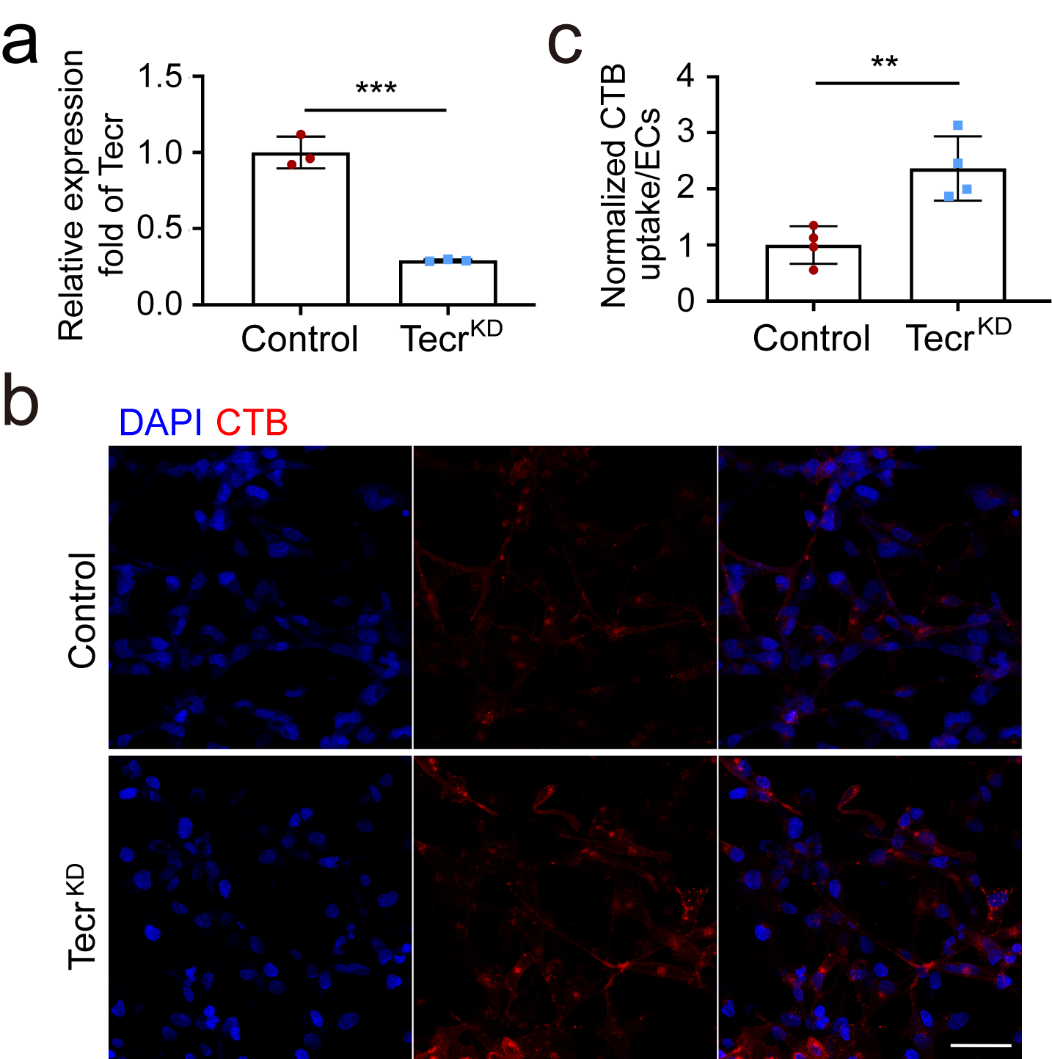


**Fig. S10. Knockdown of Tecr in hCMECs significantly enhanced uptake activity of CTB.**

a. RT-qPCR analyzed the expression of Tecr in Tecr^KD^ hCMECs. The knockdown efficiency of Tecr was 70%. n = 3 per group. *p* = 0.0003, Unpaired t test.

b. Tecr^KD^ hCMECs showed significantly enhanced uptake activity of CTB compared with control. CTB, red; DAPI, blue. Scale bars, 50 μm.

c. Quantitation of CTB uptake per cell. n = 4 per group. Data are expressed as mean ± SEM. *p* = 0.0063, Unpaired t test.


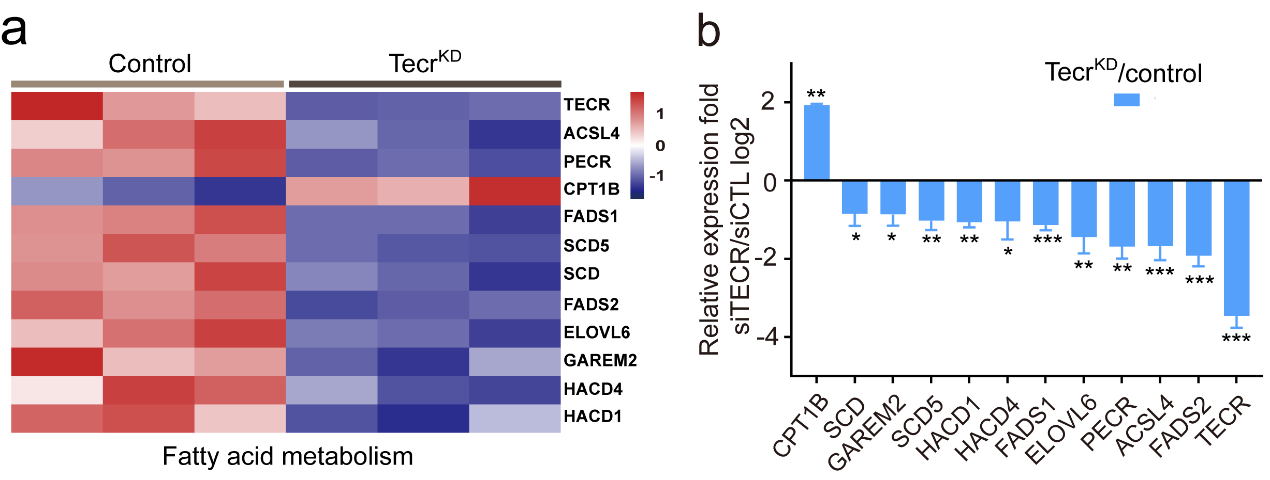


**Fig. S11.** **Knockdown of Tecr significantly altered the expression pattern of lipid metabolism pathway.**

a. The expression of desaturase and extenase was significantly decreased in Tecr^KD^ ECs. n=3 per group.

b. FAs metabolism-related genes from Transcriptome analysis were reconfirmed by RT-qPCR.

^***^*p* < 0.001, ^**^ *p* < 0.01, ^*^ *p* < 0.05. Data are expressed as mean ± SEM. Unpaired t test.


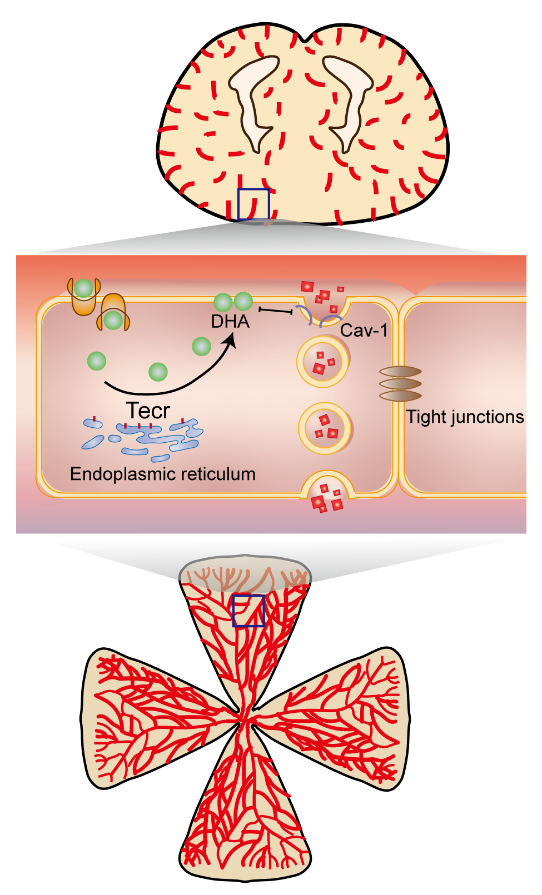


**Fig. S12. Model for the Suppression of Caveolae-Mediated Transcytosis via Regulated DHA-containing in phospholipid at the ECs of BRB/BBB.**

Tecr is expressed in the endoplasmic reticulum. Tecr as a lipid metabolic enzyme catalyzes the FAs elongation cycle and involves in both saturated and unsaturated VLCFAs, including DHA-containing species. The increased levels of DHA, and presumably other lipid changes, alter the plasma membrane composition such that caveolae vesicles are unable to form and act as transcytotic carriers. Thus, retina and brain ECs display low levels of caveolae vesicles. This suppression of caveolae formation and trafficking subsequently ensures BBB integrity under normal conditions.
